# Multi-Sensor-Assisted Low-Cost Indoor Non-Visual Semantic Map Construction and Localization for Modern Vehicles

**DOI:** 10.3390/s24134263

**Published:** 2024-06-30

**Authors:** Guangxiao Shao, Fanyu Lin, Chao Li, Wei Shao, Wennan Chai, Xiaorui Xu, Mingyue Zhang, Zhen Sun, Qingdang Li

**Affiliations:** 1 College of Electromechanical Engineering, Qingdao University of Science and Technology, Qingdao 266061, China; 2 College of Sino-German Institute Science and Technology, Qingdao University of Science and Technology, Qingdao 266061, China; 3 Haier College, Qingdao Technical College, Qingdao 266555, China; 4 College of Automation and Electronic Engineering, Qingdao University of Science and Technology, Qingdao 266061, China; 5 College of Information Science & Technology, Qingdao University of Science and Technology, Qingdao 266061, China

**Keywords:** indoor localization, multi-sensor fusion, non-visual semantic landmark, semantic map construction

## Abstract

With the transformation and development of the automotive industry, low-cost and seamless indoor and outdoor positioning has become a research hotspot for modern vehicles equipped with in-vehicle infotainment systems, Internet of Vehicles, or other intelligent systems (such as Telematics Box, Autopilot, etc.). This paper analyzes modern vehicles in different configurations and proposes a low-cost, versatile indoor non-visual semantic mapping and localization solution based on low-cost sensors. Firstly, the sliding window-based semantic landmark detection method is designed to identify non-visual semantic landmarks (e.g., entrance/exit, ramp entrance/exit, road node). Then, we construct an indoor non-visual semantic map that includes the vehicle trajectory waypoints, non-visual semantic landmarks, and Wi-Fi fingerprints of RSS features. Furthermore, to estimate the position of modern vehicles in the constructed semantic maps, we proposed a graph-optimized localization method based on landmark matching that exploits the correlation between non-visual semantic landmarks. Finally, field experiments are conducted in two shopping mall scenes with different underground parking layouts to verify the proposed non-visual semantic mapping and localization method. The results show that the proposed method achieves a high accuracy of 98.1% in non-visual semantic landmark detection and a low localization error of 1.31 m.

## 1. Introduction

As the automotive industry evolves, modern vehicles have transformed into intelligent transportation units. For continuous localization and navigation in indoor and outdoor environments, indoor location-based services (ILBSs) based on indoor mapping and localization have become a focal point of research in academia and the industry [1]. However, traditional indoor mapping approaches require human labor, low informatization level, time consumption, and high cost. Moreover, they are often restricted by various factors (e.g., privacy and vested interests) [2], making it challenging to obtain digital models of buildings. Consequently, integrating data from multiple sensors from smart devices for indoor mapping has gained favor among experts and scholars.

In recent years, indoor mapping and localization methods based on multi-sensor fusion have rapidly developed. With the assistance of a variety of sensors (e.g., cameras [3], Light Detection and Ranging (LiDAR) [4], inertial measurement unit (IMU) [5], Wireless Fidelity (Wi-Fi) [6], and ultra-wideband (UWB) [7]), innovative mobile platforms, including mobile robots, smartphones, and intelligent vehicles/autonomous vehicles, exhibit strong environmental perception capabilities. They can capture the distribution of features, such as semantic markers and wireless signals in unknown indoor environments since a user’s trajectory is related to many types of information, including visual landmarks, Wi-Fi/Bluetooth fingerprints, and more. To reduce the defects of a single sensor, a variety of multi-sensor fusion-based indoor mapping solutions have been proposed by experts and scholars.

Currently, the widely used simultaneous localization and mapping (SLAM) approaches include LiDAR-based SLAM [4], vision-based SLAM [8], and feature-based SLAM [6]. Although LiDAR-based and vision-based SLAM have promising mapping results in previous research works, they are based on the assumption that the scenes are static or the dynamic elements constitute only a small proportion of the scenes [9]. However, underground parking environments are filled with ever-changing dynamic elements, such as pedestrians, vehicles, cargo, lighting conditions, etc. These dynamic elements vary in size, shape, and speed. This usually leads to feature-matching errors, which affect the localization accuracy [8,10,11]. In addition, LiDAR SLAM or visual SLAM not only has strict requirements for sensors and computing power but also cannot be used across platforms. Fingerprinting-based indoor localization methods (e.g., Wi-Fi and Bluetooth) play a crucial role in assessing landmark similarity [12,13]. Still, because some indoor environments may not have signals and the localization accuracy needs to be improved, fingerprinting-based methods cannot be used as a standalone method for high-precision localization. Nevertheless, low-level feature-matching localization algorithms are time-consuming and have poor real-time performance. Therefore, it is necessary to construct semantic venue maps suitable for localization using high-level semantics.

With the development of sensors, mobile communication, and Internet of Things (IoT) technology, modern vehicles have become increasingly powerful in computation, interaction, communication, and perception. Now, most modern vehicles come equipped with a variety of sensors with essential localization functions, including an Accelerometer (ACC), Gyroscope (GYRO), Wi-Fi, Bluetooth, Global Navigation Satellite System (GNSS), and more. With the widespread use of modern vehicles, especially for accessing indoor parking facilities, mapping and localization methods based on modern vehicles have become a valuable research direction. The indoor mapping and localization method can be widely applied in indoor road navigation, autonomous driving (such as automated valet parking and vehicle summoning), emergency response, and other fields [14].

Currently, there are indoor map construction and localization methods based on visual and LiDAR, but these methods are only suitable for intelligent vehicles equipped with high-precision sensors such as cameras and LiDAR, lacking general applicability. Considering the impact of dynamic factors in indoor environments and the diversity of sensors equipped on modern vehicles, this paper proposes a low-cost indoor non-visual semantic map construction method based on ordinary modern vehicles. This method aims to solve modern vehicles’ mapping and localization in unknown indoor environments; the constructed non-visual semantic map can be used in future crowdsourcing mapping and also to meet the location service needs of smartphones within the indoor environment in the future. The non-visual semantic landmarks in this work are innovatively defined as the meaningful waypoints on the vehicle’s trajectory, such as entry/exit, slope entry/exit, and road node (i.e., intersection points of roads). Then, a sliding window-based semantic detection method is introduced to detect the non-visual semantic landmarks. This method uses the mileage as the window size, extracts multidimensional signal features from sensors, and detects the non-visual semantic landmark according to the signal features and their changes at window joint points (i.e., points where two sliding windows connect) or in the window. We use a sliding window-based fusion algorithm to process Wi-Fi signals to improve the confidence and stability of Wi-Fi fingerprints. Finally, we associate semantic landmarks and Wi-Fi fingerprints with waypoints to construct a lightweight trajectory non-visual semantic map of the venue.

To estimate the vehicle’s position in the venue map (i.e., the constructed trajectory non-visual semantic map), we propose a landmark matching-based localization method assisted with graph optimization. The novel landmark matching approach uses the geometry relationship between non-visual semantic landmarks to iteratively match semantic landmarks between the new trajectory map (i.e., the new trajectory map built online for positioning) and the venue map. Then, the matching relationship of map components in the map optimization window is used to update the location of each component in the new trajectory map on the venue map, and the graph optimization algorithm is applied to enhance the localization performance further.

In summary, the main contributions of this paper are as follows:

(1) For mapping in unknown indoor environments with a modern vehicle, a non-visual semantic landmark detection and non-visual semantic map construction method is proposed. The lightweight semantic map consists of waypoints, Wi-Fi fingerprints, and non-visual semantic landmarks.
(2) To accurately estimate the location of modern vehicles on the venue map, a feature-matching-based localization method is proposed. The geometry relationship between non-visual semantic landmarks is used for iterative landmark matching. The graph optimization algorithm is utilized to enhance the positioning accuracy of modern vehicles on indoor semantic maps.
(3) The proposed non-visual semantic map construction and localization methods are experimentally validated, demonstrating their effectiveness in addressing low-cost indoor localization and navigation issues for modern vehicles, especially in scenarios of indoor parking lots.

## 2. Related Works

### 2.1. Semantic Detection and Map Construction

Sensor data for environmental perception are essential in constructing semantic maps [15]. Currently, sensors such as LiDAR and cameras in single-device platforms are commonly used to build semantic maps. The widely used cameras include monocular cameras, stereo cameras, and RGB-D cameras. LiDAR provides high-frequency, long-range, and centimeter-level high-precision measurements, unaffected by lighting conditions, and requires relatively low computational performance. The main LiDAR-based SLAM approaches include filter-based SLAM (such as particle filters and extended Kalman filters (EKFs)) [16,17,18,19,20], and graph optimization-based SLAM (such as least squares, factor graphs, and so on) [21,22,23]. However, LiDAR-based SLAM is unsuitable for large open environments and utilizes high-cost sensors. Moreover, the constructed maps lack semantic information.

Compared to LiDAR, visual-based SLAM can extract more semantic information from images and is applicable to a wider range of scenarios, while the sensors used in these methods are relatively low-cost. For example, the visual SLAM can form grayscale or color images compared to the point cloud acquired by the LIDAR SLAM [21]. Main visual SLAM methods include direct method [24] (e.g., DSO [25,26], SVO [27], VIO [28]) and feature-based methods (e.g., DP-SLAM [8], ORB-SLAM2 [29]). In our team’s prior research, Chai et al. presented a vanishing point-assisted VI-SLAM in 2021, utilizing vanishing points to reduce drift errors of the SLAM system and improve the pedestrian’s trajectory estimation accuracy [30]. Subsequently, Li et al. proposed VI-SLAM based on deep learning and spatial constraints in [31], aiming to distinguish dynamic and static semantic targets in the scene. However, visual SLAM is inherently affected by the limitations of visual sensors. Firstly, monocular, stereo, and depth camera systems are sensitive to environmental lighting and optical textures. Secondly, captured images may lack texture and can become blurry when the platform moves at high speeds [32,33]. Moreover, the visual SLAM does not need depth information but does require powerful GPU support. These weaknesses often limit the industrial application of visual SLAM. In 2019, Chai et al. proposed methods to identify and update non-visual semantic landmarks on the vehicle’s trajectory (also known as trajectory landmarks) for the first time and published the corresponding patents [34,35]; the related technology has already been applied in some new types of intelligent vehicles to assist indoor automated valet parking.

### 2.2. Indoor Localization

As a core component of location-based services (LBSs), indoor localization garnered significant attention from scholars worldwide. Many researchers advocated combining Recurrent Neural Networks (RNNs) with sensor data to address indoor positioning challenges. In reference [36], the magnetic-based localization approach was viewed as an approximation problem of recursive functions. They trained Long Short-Term Memory Networks (LSTMs) using time-series magnetic field data created through dual-sliding windows to determine the user’s location. Shu [37] introduced directional information in the fingerprint construction and localization process, achieving accurate positioning results using multiscale RNNs and ensemble learning mechanisms. However, collecting magnetic feature datasets or sequences of magnetic field signals in advance entails substantial human and time costs, making it less feasible for large-scale deployment.

Wi-Fi-based localization methods could achieve meter-level location accuracy in indoor environments with high adaptability and low cost. This approach comprises two main methods: triangulation and fingerprinting [38,39]. Triangulation relies on pre-acquiring the coordinates of Wi-Fi transmitters, and it is sensitive to environmental factors. Consequently, the fingerprinting approach became the mainstream approach, such as nearest neighbor techniques [40,41] (e.g., K-nearest neighbor, WKNN) and maximum likelihood probability techniques. In previous research by our team, Chai presented a landmark matching location method that fuses Wi-Fi, PDR, and visual semantic information [42]. This method achieved low positioning error (less than half a meter) in office building scenes for a single-trajectory semantic map. Moreover, the graph optimization algorithm was introduced in our work [43] to further enhance localization accuracy. Reference [44] proposed a novel location method based on local and global node similarity, aiming to reduce storage space while preserving node information.

Inspired by the work described in [42,43,44], this paper constructs a lightweight semantic map that includes non-visual semantic landmarks, Wi-Fi fingerprints, and waypoints. Based on this foundation, a landmark (non-visual semantic and Wi-Fi fingerprints) matching-based graph optimization localization algorithm is proposed.

## 3. Semantic Map Construction and Indoor Localization

**Figure 1:**
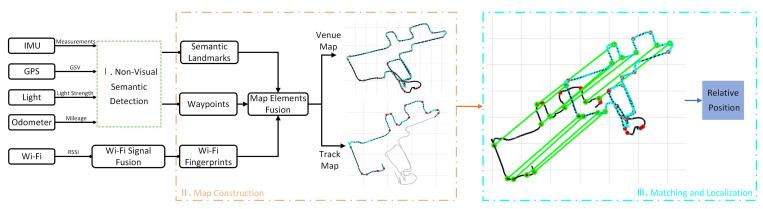
An illustration of the proposed system structure. I. Non-visual semantic detection, II. map construction, and III. matching and localization (the black, red and cyan dots represent the waypoint, non-visual semantic Landmark and Wi-Fi fingerprint, and the green lines represent the matching relationship of non-visual semantics between the venue map and the trajectory map).

The map construction and localization system proposed in this paper is illustrated in Figure 1. The first part involves multi-sensor-assisted non-visual landmark detection, utilizing IMU measurements, GNSS data, light sensor data, and odometer data as inputs. This paper employs a sliding window-based non-visual semantic landmark detection algorithm to identify non-visual semantic landmarks. The second part focuses on constructing a single-trajectory semantic map, encompassing waypoints, non-visual semantic landmarks, and Wi-Fi fingerprints. The third part introduces a landmark matching-based localization method assisted with graph optimization, enabling the intelligent vehicle to determine its position in the preconstructed semantic map. 

### 3.1. Non-Visual Semantic Landmark Detection

The partial landmark information used for constructing trajectory maps needs to be identified by using motion and localization sensors. These landmarks are known as non-visual semantic landmarks and serve as crucial references for subsequent vehicle trajectory matching and location. As non-visual semantic landmark is associated with real-time mapping and localization, it must meet the following criteria:

i. It should be reproducible during localization and navigation in the mapping venue;
ii. It can be detected by low-cost inertial and localization sensors with low computational requirements;
iii. The quantity and quality of non-visual semantic landmarks should be sufficient for mapping and localization.

To achieve the above criteria, we analyzed widely used sensors mounted on modern vehicles, such as ACC, GYRO, GNSS receiver, light sensor, and odometer. As a result, we classified non-visual semantic landmarks as entry/exit, slope entry/exit, and road node. The corresponding sensors, key features, and auxiliary features used for non-visual semantic landmark detection are summarized in Table 1.

#### 3.1.1. Data Preprocessing

Before semantic landmark detection, this paper preprocesses raw measurements collected by the inertial sensors (GYRO and ACC), GNSS receiver, odometer, and light sensor. Specifically, to standardize the data format, all data are synchronized using a time synchronization table to unify the time to Coordinated Universal Time (UTC) and assign the odometer data for synchronized data. The speed estimated by the vehicle odometer is used as an external observation to correct the speed estimation of IMU, producing the accurate vehicle dead reckoning (VDR) trajectory. Additionally, data from the ACC, GYRO, light intensity, and Global Satellite Visibility (GSV) are extracted to reduce data redundancy. It is important to note the processing and extraction of GSV data. Since vehicles typically receive signals from multiple satellites during operation, further processing is necessary for accurate entrance and exit identification. Elevation angles and signal strength data of each satellite in each frame of the GSV signal are extracted. Satellites with elevation angles exceeding a predetermined threshold (high elevation angles) are selected as valid satellite data. The composite signal strength ($snr_{jj,m}$) for each frame is then calculated by weighted averaging the signal strengths $snr_{jj,ii}$ from the valid satellites. The formula for $snr_{jj,m}$ is defined as follows:

(1) $$snr_{jj,m}=\frac{\sum \left(snr_{jj,ii}\times \sin\left(elv_{jj,ii}\right)\right)}{\sum \sin\left(elv_{jj,ii}\right)}$$

In (1), $snr_{jj,ii}$ and $elv_{jj,ii}$ represent the signal strength and corresponding elevation angle of the (ii)th valid satellite in the (jj)th frame, respectively. 

#### 3.1.2. Semantic Landmark Detection

**Figure 2:**
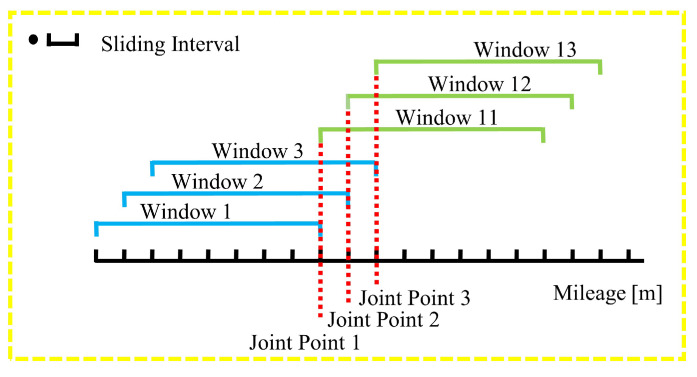
Diagram of joint points.

To detect entry/exit and slope entry/exit, this paper introduces the concept of joint points within sliding windows, as illustrated in Figure 2. To maintain the spatial consistency of various signal features and to meet the detection needs of different non-visual semantics, multiple sliding windows are created with distance scale. The signal features within each sliding window are computed. When joint points between windows are generated, they are extracted, and the changes in various features between the two windows adjacent to the joint points are calculated. These feature change values are then input into a Spark logical regression model that has been trained with various semantic features and labeled data. The model calculates the confidence level of the joint points as potential semantic landmarks. Based on a predefined confidence threshold, candidate joint points for different types of landmarks are initially selected. Subsequently, landmark selection windows are extracted according to the positions of all candidate joint points in the trajectories. Within these windows, the candidate with the highest confidence is chosen as the specific type of trajectory landmark, and landmark data (e.g., type, attributes, and mileage) are computed.

**Figure 3:**
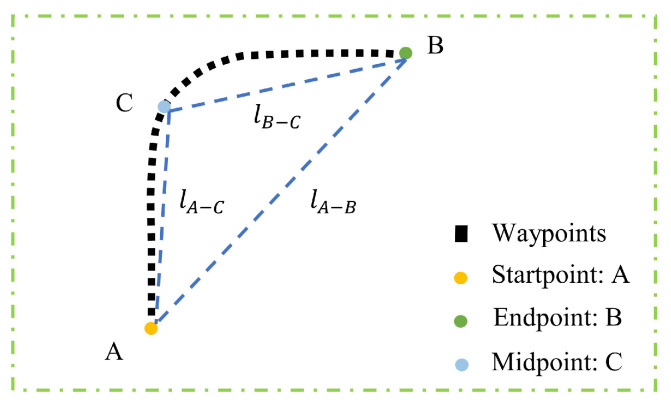
The diagram of the road node’s features in a sliding window (the blue dashed line is the auxiliary line).

The detection of road nodes varies slightly from the semantic detection described above. The detection of road nodes primarily depends on the changes in heading angle △ψ, trajectory curvature κ, and the aspect ratio γ of triangles (formed by the start, middle, and end points of a sliding window) within a sliding window. The midpoint of the window serves as the candidate point for node landmarks. The diagram of the road node’s features in a sliding window is shown in Figure 3.

(2) $$\begin{array}{l} \Delta \psi =mean\left(\psi_{A-C}\right)-mean\left(\psi_{C-B}\right);\\ \kappa =\frac{l_{A-C}\times l_{B-C}\times l_{A-B}}{S_{\Delta ABC}};\\ \gamma =\frac{l_{A-B}}{l_{A-C}} \end{array}$$

In (2), $\psi_{A-C}$ and $\psi_{C-B}$, respectively, represent the heading angles of each waypoint between point A and point C and from point C to point B.

### 3.2. Single-Trajectory Semantic Map Detection

#### 3.2.1. Wi-Fi Fingerprint Collection

Wi-Fi fingerprints are collected simultaneously when the vehicle is moving along the trajectory of mapping or localization. To mitigate the impact of non-line-of-sight propagation, co-channel interference, and mobile access points on the received signal strength of an access point (AP), this paper employs a sliding window-based Wi-Fi fingerprint fusion method to enhance the stability of Wi-Fi fingerprints [42]. The timestamp of fused Wi-Fi fingerprints is updated as the mean of all the fingerprints within the sliding window. The maturity of fused APs is the number of occurrences of that AP in the sliding window. The received signal strength (RSS) of fused AP is calculated as the average of that AP within the sliding window. The fused APs are sorted by the RSS.

#### 3.2.2. Map Construction

**Figure 4:**
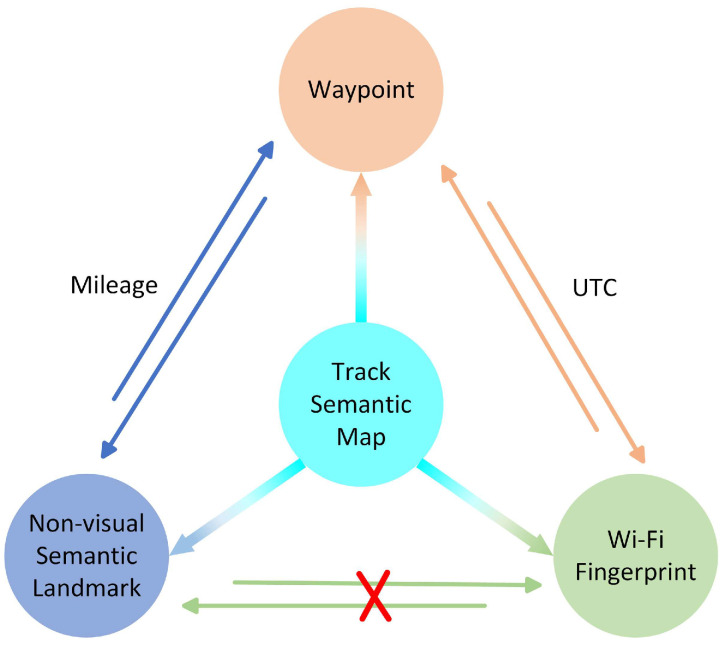
The association relationship among the waypoints, non-visual semantic landmarks, and Wi-Fi fingerprints in a single-trajectory semantic map.

The single-trajectory semantic map serves as a prerequisite for subsequent vehicle localization. This map consists of feature units (e.g., waypoints, non-visual semantic landmarks, and Wi-Fi fingerprints) and their feature information, including maturity, confidence, signal strength, coordinate, and heading angle. Figure 4 illustrates the relationships among these feature units in a single-trajectory semantic map. In the constructed single-trajectory semantic map, waypoints are associated with Wi-Fi fingerprints through UTC, while trajectory semantic landmarks are linked to waypoints according to mileage features. However, due to the lack of strong direct associations between non-visual semantic landmarks and Wi-Fi fingerprints, they cannot be directly associated. To reduce data redundancy and achieve map lightweight, only waypoints, non-visual semantic landmarks, and Wi-Fi fingerprints are retained in the map file.

### 3.3. Localization

#### 3.3.1. Landmark Matching

A single-trajectory semantic map of a specific scene is constructed and used as a venue map for localization. When the intelligent vehicle is near the parking area, it starts to acquire sensor data. The real-time construction of the trajectory map for a modern vehicle is achieved using the mapping method. To achieve continuous outdoor and indoor localization, this paper utilizes a landmark matching-based localization method assisted with graph optimization to estimate the vehicle’s location in the built venue map. The proposed localization algorithm includes two phases: initial matching localization and secondary matching localization.

In the initialization matching phase, if there are semantic landmarks in the sliding window of the trajectory map, preliminary matching of non-visual semantic landmarks is performed according to their types. Otherwise, only Wi-Fi fingerprinting is conducted. Multiple semantic landmarks in the venue map may correspond to the unmatched landmarks in the trajectory map. Therefore, one-to-one correspondence of the non-visual semantic landmarks between the trajectory and venue map is realized by Wi-Fi fingerprinting. It is important to note that this phase needs to complete the matching for landmarks since it serves in the secondary matching localization.

In this phase, the landmark matching quality $Score_{M}$ is defined as follows:

(3) $$Score_{M}={\left(\mu_{N}\times Dis_{N-N}+\mu_{W}\times Dis_{W-W}\right)}^{-1}$$

In (3), $Dis_{N-N}$ and $Dis_{W-W}$ denote the distances of matched non-visual semantic landmarks and Wi-Fi fingerprints between the new trajectory map and the scene map. $\mu_{N}$ and $\mu_{W}$ represent the weights. $Dis_{W-W}$ directly reflects the degree of Wi-Fi fingerprint matching and is composed of a weighted combination of the Euclidean distance ($Dis_{euc}$) and sequence distance ($Dis_{seq}$) of Wi-Fi fingerprints, and it is defined as follows:

(4) $$Dis_{W-W}=\omega_{euc}\times Dis_{euc}+\omega_{seq}\times Dis_{seq}$$

In the secondary matching localization phase, non-visual semantic landmark matching becomes the primary method since Wi-Fi equipment may be absent in indoor underground parking areas. The rules for non-visual semantic matching in this phase rely on the correlation between the semantic landmarks to be matched and the already matched semantic landmarks, primarily considering height, angle, and distance relationships.

Assuming that there are V non-visual semantics in the scene map, and there are already m(m≥n) semantics matched between the new trajectory map and the venue map, $S_{t,i}$ and $S_{v,i}$ represent the (i)th matched non-visual semantics in the trajectory map and the venue map, respectively. The matching rule for the (m+1)th non-visual semantic landmark is defined as follows:

(5) $$\min\left(\begin{array}{l} \varsigma_{P}\left(\sum_{i=m-n+1}^{m}\left(P_{t,m+1}-P_{t,i}\right)-\sum_{i=m-n+1}^{m}\left(P_{v,j}-P_{v,j}\right)\right)+\\ \varsigma_{\theta }\left(\sum_{i=m-n+1}^{m}\left(\theta_{t,m+1}-\theta_{t,i}\right)-\sum_{\mathrm{ii}=m-n+1}^{m}\left(\theta_{v,j}-\theta_{v,j}\right)\right)+\\ \varsigma_{h}\left(\sum_{i=m-n+1}^{m}\left(h_{t,m+1}-h_{t,i}\right)-\sum_{i=m-n+1}^{m}\left(h_{v,j}-h_{v,j}\right)\right) \end{array}\right),\left(j\in V\right)$$

In (5), P, θ, and h represent the position, angle, and height of the non-visual semantic landmarks, respectively, and $\varsigma_{P}$, $\varsigma_{\theta }$ and $\varsigma_{h}$ are the corresponding weights, respectively.

#### 3.3.2. Graph Optimization-Based Localization

While ensuring the association relationship between components (non-visual semantic landmarks, Wi-Fi fingerprints, and waypoints) in each map and matching relationship between maps, we employ a graph optimization-based method to maximize the alignment between the trajectory map and venue map, thereby reducing location errors. When constructing the graph optimization model, this paper uses associations as edges and considers waypoints, non-visual semantic landmarks, and Wi-Fi fingerprints as vertices. We find a matrix $\beta_{f}$ through the association relationship and the matching relationship, which minimizes the loss function $f_{err}$, and then the Gauss–Newton method is used to achieve the optimization purpose:

(6) $$\beta_{f+1}=\beta_{f}-H^{-1}*\nabla f_{err}$$

In (6), $\nabla f_{err}$ represents the gradient vector of the loss function $f_{err}$ at $\beta_{f}$, and H is the Hessian matrix of the loss function $f_{err}$.

During localization, the landmarks within the optimization window of the trajectory map are used as target landmarks to match with the venue map. This process calculates the rotation and translation matrix and updates the positions of waypoints, semantic landmarks, and Wi-Fi landmarks on the map using the graph optimization method. After the rotation and translation, the position $P^{t}$ of the intelligent vehicle platform on the indoor semantic map is defined as follows:

(7) $$P^{t}=T^{t}P^{E}$$

In (7), $T^{t}$ represents the rotation and translation matrix of the local map, and $P^{E}$ represents the original position coordinates of the intelligent mobile platform on the local map.

## 4. Experiments

This section first describes the experimental equipment and test fields and then presents the results of the field experiments.

### 4.1. Experiments Setup

**Figure 5:**
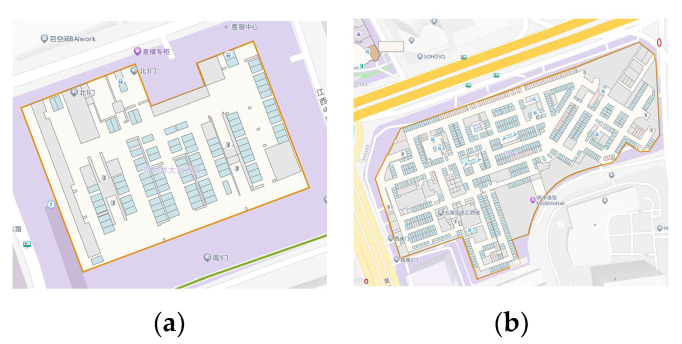
A schematic diagram of the experimental scene. (**a**) is a schematic of the floor B3 in the mall 1 scene and (**b**) is a schematic of the floor B4 in the mall 2 scene.

To verify the effectiveness of the mapping and localization system proposed in the previous sections, we selected the AITO M5 Standard Edition intelligent vehicle platform as the test device, which is equipped with a BOSCH SMI240 IMU, a u-blox NEO-M8Q-10A GNSS module, an Espressif ESP32-S2 Wi-Fi module, and an ams-osram SFH 5711-2/3-Z light sensor. The basic parameters of the SMI240 IMU are range: ±300°/s (Ω), ±16 g (a); gyro offset error: ±5°/s. The experiments were conducted in two shopping malls located in Shanghai as shown in Figure 5. The tester drove the modern vehicle from the outdoors, entering the indoor parking lot through the same entrance, simulating the typical user experience of navigating within an indoor parking lot. Subsequently, they exited through the same exits. This experiment was conducted six times, with three repetitions for each scene. The intelligent vehicle traveled distances of 1091.7 m in mall 1 and 1136.7 m in mall 2. While the routes in each scene remained consistent, there were variations in driving time and vehicle speed. Due to the differences in the sensors configured in different types of modern vehicles, to realistically simulate the system’s performance across various types of modern vehicles, we used the odometer-optimized vehicle’s trajectory map as the venue map for both scenes and did not use the odometer-optimized trajectory map for localization.

### 4.2. Non-Visual Semantic Landmark Detection Result

The choice of the sliding window size significantly impacts the effectiveness and precision of non-visual semantic landmark detection. Therefore, the selection of the window scale should meet two criteria: (i) the precision requirements for detection and (ii) the recognition needs of different types of non-visual semantic landmarks. Thirty sets of data were respectively employed to train Spark logistic regression models for the recognition of entry/exit, slop entry/exit, and road node. The semantic detection rate and precision were determined by contrasting them with video image timestamps. The results of the semantic detection effectiveness, compared with the ground truth, are presented in Table 2.

### 4.3. Localization in Venue Map

**Figure 6:**
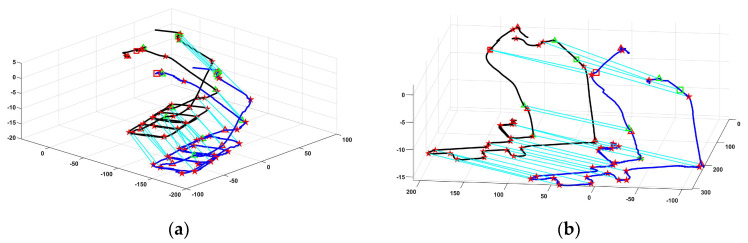
Non-visual semantic landmark matching results between the trajectory map and the venue map in two mall scenes. (**a**,**b**) represent the results of non-visual semantic landmark matching in mall 1 and mall 2. Red star represents road node, green and red triangle represent slop entry/exit, green and red square represent entry/exit. Black line indicates the waypoints of the constructed scene map, the blue line indicates the waypoints of the new localization map, and the cyan line represent the matching relationship of non-visual semantics between the venue map and the new localization map.

**Figure 7:**
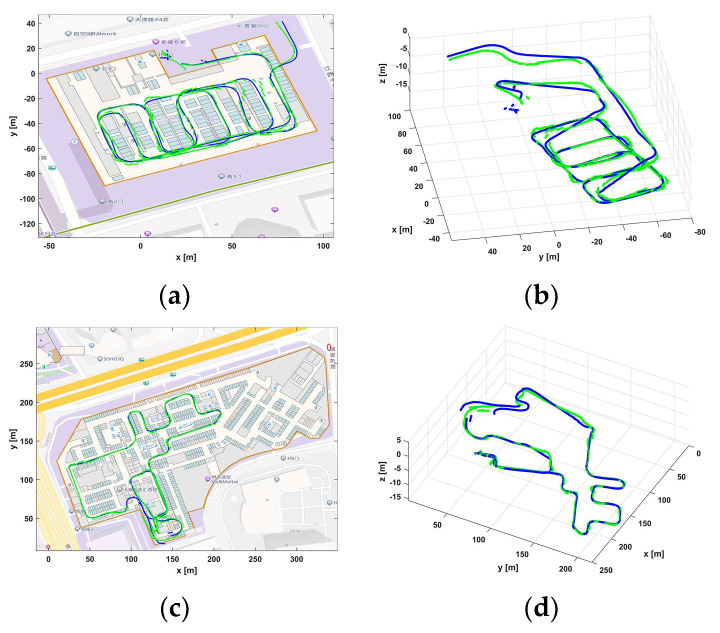
The localization results based on non-visual semantic landmark matching. (**a**,**b**) represents a schematic diagram of 2D and 3D localization results in mall 1. (**c**,**d**) represents a schematic diagram of 2D and 3D localization results in mall 2. The blue line indicates the constructed scene map, and the green line indicates the new localization map.

To estimate the vehicle’s location in the constructed map and validate the effect of the proposed landmark matching-based graph optimization localization method in the underground parking, this paper set the requirement to complete the matching of the first 4 semantics in the trajectory map during the initialization location phase. The non-visual semantic matching result between the trajectory map and the venue map is shown in Figure 6. The black line indicates the venue map, the blue line indicates the trajectory map, and the green line indicates the matching relationship between the non-visual semantics. Figure 7 shows the final localization result based on landmarks matching. Please note that due to factors such as errors in trajectory estimation and mapping during the map construction, there are slight misalignments between the semantic map and the background map provided by AMAP.

**Figure 8:**
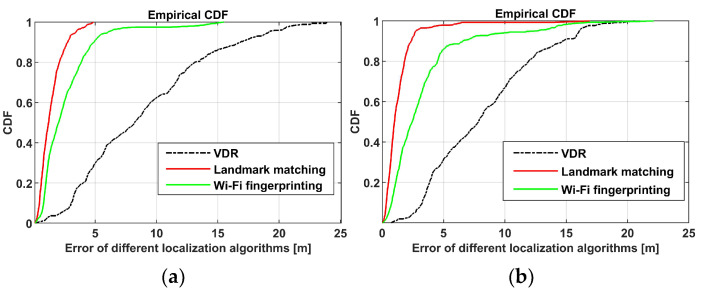
CDF of localization errors of two malls. (**a**) is CDF of location errors of mall 1 scene, and (**b**) is CDF of location errors of mall 2 scene.

The approximate locations of the trajectory map relative to the semantic landmarks in the non-visual semantic map were compared to the ground truth locations, and cumulative distribution functions (CDFs) of localization errors were calculated. Compared with the Wi-Fi fingerprinting-based localization, the landmark matching-based localization method has higher localization accuracy, as shown in Figure 8. From the mall 1 scene, the average localization error of the proposed landmark matching-based localization method is 1.41 m. Meanwhile, the average localization error of the Wi-Fi fingerprinting-based localization method is 2.62 m. Additionally, in the mall 2 scene, the average localization error of the proposed landmark matching-based localization method is 1.34 m, representing an improvement of 59.64% compared to the Wi-Fi fingerprinting-based localization method.

## 5. Conclusions

This paper proposes a map construction and location system for unknown indoor environments. The system relies on the IMU, odometer, light sensor, Wi-Fi receiver, and GNSS receiver equipped with the modern vehicle. To reduce the adverse impact of dynamic factors on the semantic map, this paper uses the novel non-visual semantic landmark based on fixed position to build a semantic map, uses a sliding window-based method to detect the non-visual semantic landmark, and then builds a lightweight indoor non-visual semantic map that includes waypoints, semantic landmarks, and Wi-Fi fingerprints. To estimate the position of the modern vehicle in the venue map, the localization method realizes the matching of non-visual semantic landmarks between the trajectory map and the venue map by using the relationship of non-visual semantic landmarks. On this basis, the graph optimization method is used to further improve the localization accuracy. Finally, the performance is verified in two underground parking scenes with different layouts. The results show that the proposed method can be used to address the lost-cost indoor localization problems of various modern vehicles.

However, the single-trajectory non-visual semantic map often cannot cover all areas of the indoor scene; it is necessary to fuse a number of single-trajectory maps from multiple vehicles to build a complete indoor non-visual semantic map, namely crowdsourced mapping, which is also our current research direction. In addition, crowdsourced mapping can further enhance the maturity of Wi-Fi fingerprints and reduce detection and location errors of non-visual landmarks.

## Figures and Tables

**Table 1:** The corresponding sensors, key features, and auxiliary features used for non-visual semantic landmark detection.

Type	Key Features	Auxiliary Features	Sensors
entry/exit	GSV	Light intensity	GNSS receiver, light sensor
slop entry/exit	Pitch angle	ACC	GYRO, ACC
road node	Yaw angle	Curvature, scale	GYRO, ACC

**Table 2:** Detection effect of non-visual semantic landmarks in two mall scenes.

Mall	False Rate	Miss Rate	Error of Location (m)
mall 1	1.90%	1.90%	1.39
mall 2	0.00%	7.61%	1.89

## Data Availability

The data are contained within this article.
